# A shallow chest correlates with the aortic position in the normal spine: features resembling those observed in structural scoliosis

**DOI:** 10.1186/1748-7161-9-14

**Published:** 2014-08-30

**Authors:** Toshio Doi, Yoshihiro Matsumoto, Osamu Tono, Kiyoshi Tarukado, Katsumi Harimaya, Seiji Okada, Kensuke Kubota, Mitsumasa Hayashida, Yukihide Iwamoto

**Affiliations:** 1 Department of Orthopaedic Surgery, Kyushu University Beppu Hospital, 4546 Tsurumi, Beppu, Oita 874-0838, Japan; 2 Department of Orthopaedic Surgery, Graduate School of Medical Sciences, Kyushu University, 3-1-1 Maidashi, Higashi-ku, Fukuoka 812-8582, Japan

**Keywords:** Scoliosis, Rib cage deformity, Aorta, Shallow chest

## Abstract

**Background:**

Right thoracic curvature, rib cage deformities and aortic left shift are features of adolescent idiopathic scoliosis that are correlated with each other. We recently reported that disturbance of ribcage development results in progressive thoracic scoliosis in mice. Recently, it has been confirmed that the normal spine exhibits right thoracic curvature and rib cage deformities and that these deformities worsen during the adolescent period. The purpose of this study was to examine whether rib cage deformities correlate with thoracic side curvature in the normal spine, as observed in scoliosis, which is important basic knowledge needed to elucidate the causative factors of adolescent idiopathic scoliosis.

**Methods:**

To examine the relationship between rib cage deformities and thoracic side curvature in the normal spine, CT scans of 148 consecutive adult females were examined. The anteroposterior chest dimension, aortic location and rib cage rotation were measured on CT scans obtained at the T8 level. The thoracic side curvature (T5-T12) was also measured on chest radiographs.

**Results:**

The anteroposterior chest dimension exhibited a significant correlation with aortic left shift. The aortic location and rib cage rotation were correlated, and the rib cage rotation and thoracic side curvature were correlated.

**Conclusions:**

There was a significant correlation between a shallow chest and the aortic position, between the aortic position and the rib cage rotation and between the rib cage rotation and the thoracic side curvature in the normal spine. These findings suggest the possibility that rib cage development is one of the causative factors of adolescent idiopathic scoliosis.

## Background

Adolescent Idiopathic Scoliosis (AIS), which dramatically worsens in the adolescent period, is characterized by the features of right thoracic scoliosis, a shallow chest and aortic left shift [1-6]. The causal relationship between scoliosis and chest deformities is unknown.

Cole AA et al. reported that the anteroposterior chest dimension in thoracic scoliosis patients is significantly smaller than that observed in normal subjects [5,6]. It is known that a correlation exists between the anteroposterior chest dimension, aortic location and the severity of thoracic curvature in AIS [7]. We previously reported that a disturbance of ribcage development leads to progressive structural scoliosis in a mouse model and demonstrated that the pathomechanisms of rib cage deformities and the associated imbalanced load on the vertebral body result in structural scoliosis [8]. The mice model also demonstrated that the position of the heart is a significant factor influencing the direction of thoracic curvature.

Recently, it has been confirmed that the normal spine exhibits right thoracic curvature [9,10], vertebral rotation [11] and rib cage deformities [12] and that these deformities worsen during the adolescent period. It remains unknown whether rib cage deformities and thoracic side curvature coexist and/or are correlated with each other. If a shallow chest and the position of the heart and aorta are factors that influence the thoracic side curvature, then these correlations can be observed in the normal spine.

To examine the relationships between rib cage deformities, the aortic position and thoracic side curvature in the normal spine, CT scans of 148 consecutive females 20 to 29 years of age were examined.

## Methods

### Patients

The patients who were enrolled in this study were those who had visited our hospital to undergo chest radiography and CT scans to investigate them for other diseases. The exclusion criteria were obvious chest, spine or lung disease and a side curvature of over 10 degrees. We recruited 148 consecutive females who were 20 to 29 (average 25.8) years of age. The patient background information and underlying diseases are shown in Table 1. No new CT scan examinations were ordered for the current study. The present project received approval from the Ethical Commission of our institute.

**Table 1:** The background diseases in the patients

**Type of disease**	**N = 148**
Malignant disease	59
Collagen disease	20
Inflammatory disease	17
Infection	13
Benign tumor	11
Renal disease	10
Hepatitis	6
Gynecological disease	2
Pneumonia	2
Trauma	2
Meningitis	1
Hip necrosis	1
Labyrinthine syndrome	1
Headache	1
Spinocerebellar degeneration	1
Asthma	1
Total	148

### Measurement

The thoracic side curvature in the normal spine was measured according to Cobb’s method, as previously described [13]. Standing chest PA radiographs were obtained. One line was drawn along the superior end plate of T5 and one line was drawn along the lower end plate of T12 on a computer screen using the Fuji Synapse angle measurement system (Fuji Synapse System, Fujifilm holdings, Tokyo, Japan). If the end plate was indistinct, the line was drawn through the pedicles. A right convex curve was assigned a positive value and a left curve was assigned a negative value. Among the 148 patients, 117 patients had right convex curves (> +1 degree), 13 patients had neutral convex curves (from -1 degree to +1 degree), and 18 patients had left convex curves (< -1 degree). We could not find anything unusual about the shallow chest or aortic position in the patients with left curves compared with the patients having either right or neutral curves.

**Figure 1:**
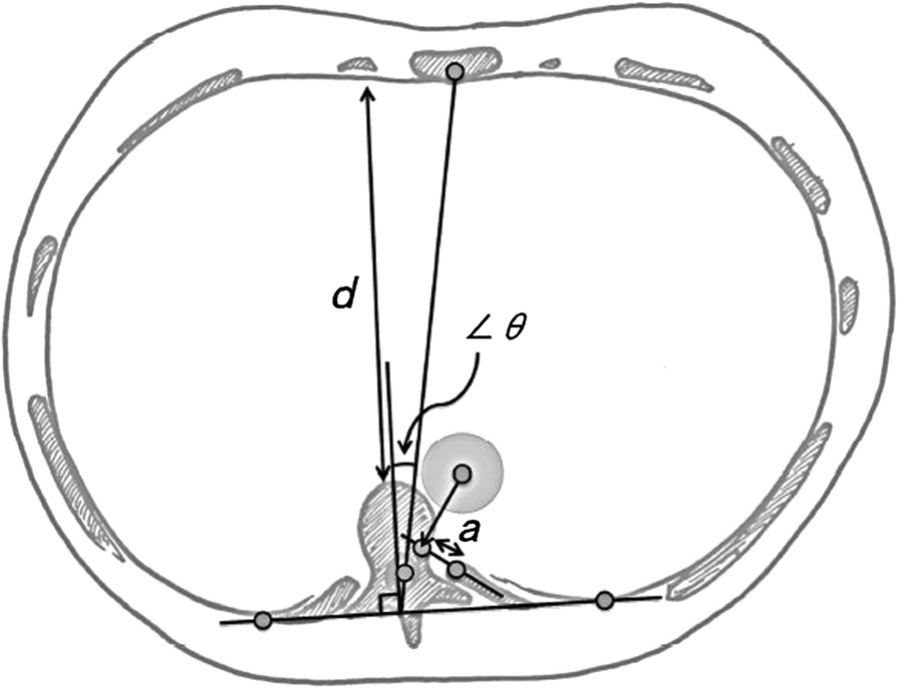
**CT image measurement.** The anteroposterior chest dimension (*d*), aortic location, whether a positive value was found on the anterior-right side (*a*) and the ribcage rotation angle (∠*θ*) were measured on CT images. Rightward ribcage rotation was defined as positive.

The CT measurements were calculated on the computer screen (Fuji Synapse System, Fujifilm holdings, Tokyo, Japan), as previously described [7]. The anteroposterior chest dimension (*d*), aortic location (*a*) and ribcage rotation angle (∠*θ*) were measured at the T8 level (Figure 1). The anteroposterior chest dimension was defined as the shortest distance between the vertebral body and the inner anterior chest wall (*d*). The aorta is usually located in front of the vertebra at the T8 level, however, in severe AIS, it sometimes shifts left-posterior and is located in front of the neck of the rib. To measure the aortic location in the normal spine, we drew a baseline in the middle of the neck of the rib and decided on the point vertically from the center of the aorta to the baseline and determined the distance (*a*) from the anterior rib head to that point (a positive value if it was found on the anterior-right side). The ribcage rotation angle (∠*θ*) was defined as the angular divergence of a line drawn at right angles to a line joining the posterior inner chest wall bilaterally, from the line joining the neural canal to the sternum. Rightward ribcage rotation was defined as positive.

The GraphPad Prism statistical software program (GraphPad Software, CA, USA) was used for the statistical analyses. The statistical methods included Spearman’s rank correlation coefficients. *P*-values of less than 0.05 were considered to be statistically significant.

## Results

**Figure 2:**
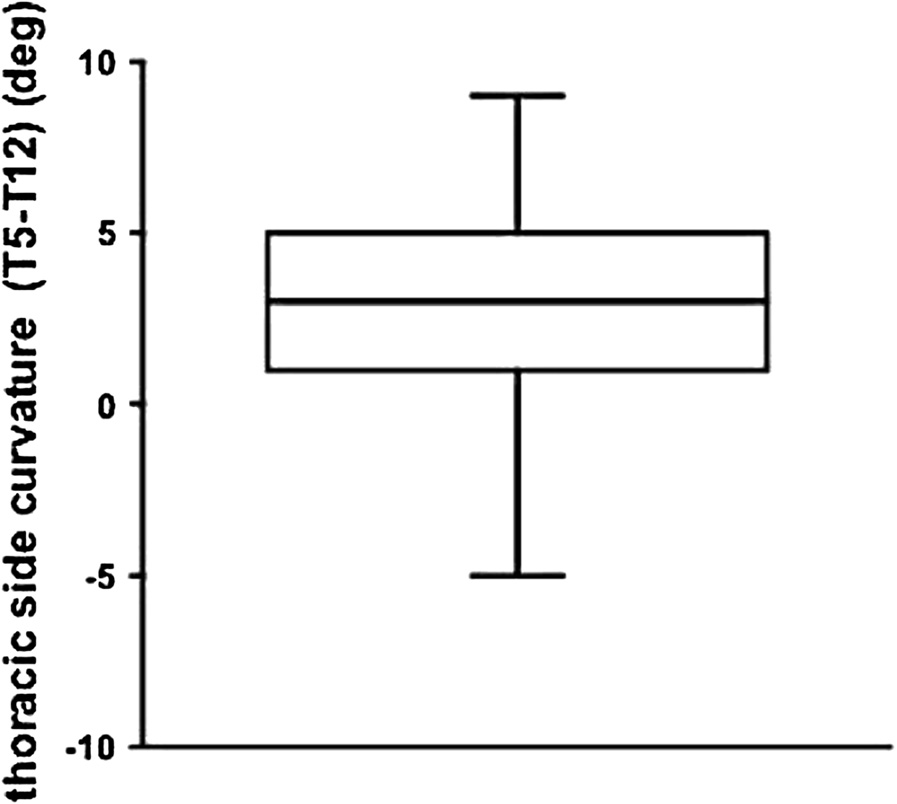
**Right thoracic curvature in the normal spine.** To evaluate the thoracic side curvature in the normal spine, Cobb’s angles were measured from T5 to T12 using standing chest radiographs. Right-sided curvature was given a positive value. The mean thoracic side curvature was 3.0 ± 3.0 (mean ± SD).

**Figure 3:**
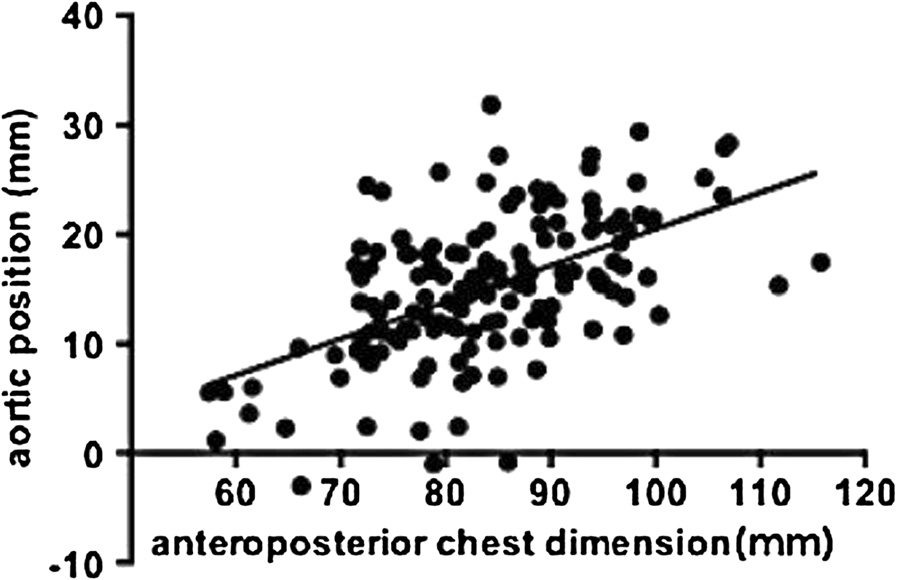
**Correlation between the anteroposterior chest dimension and aortic position.** There was a statistically significant correlation between the anteroposterior chest dimension and aortic position (ρ = 0.527, p < 0.0001, Spearman’s rank correlation coefficient).

**Figure 4:**
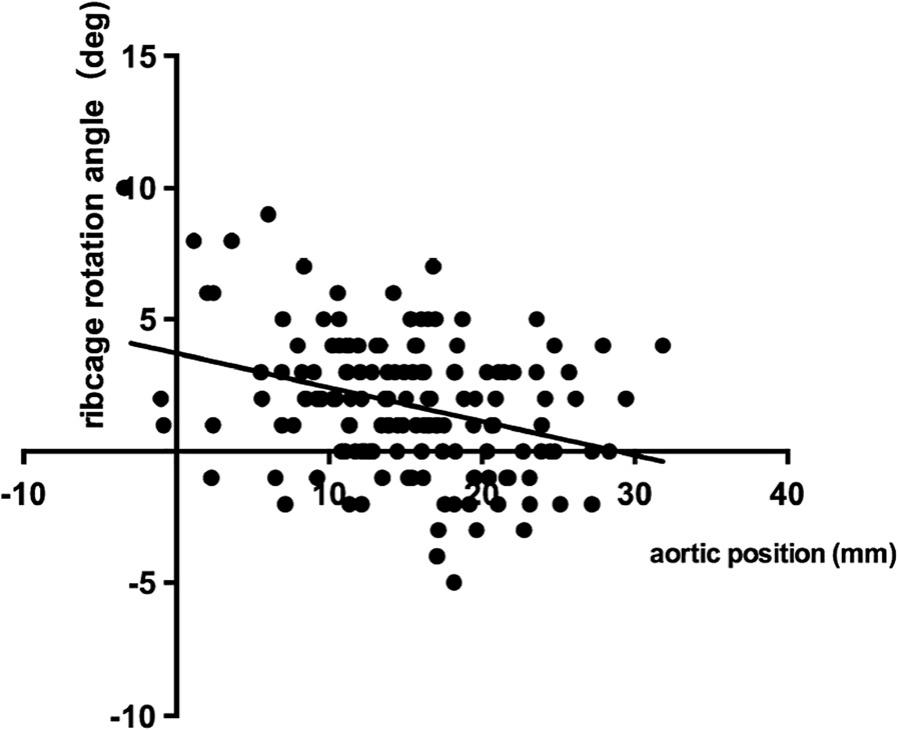
**Correlation between the aortic position and ribcage rotation angle.** There was a statistically significant correlation between the aortic position and ribcage rotation angle (ρ = -0.283, p = 0.0005, Spearman’s rank correlation coefficient).

**Figure 5:**
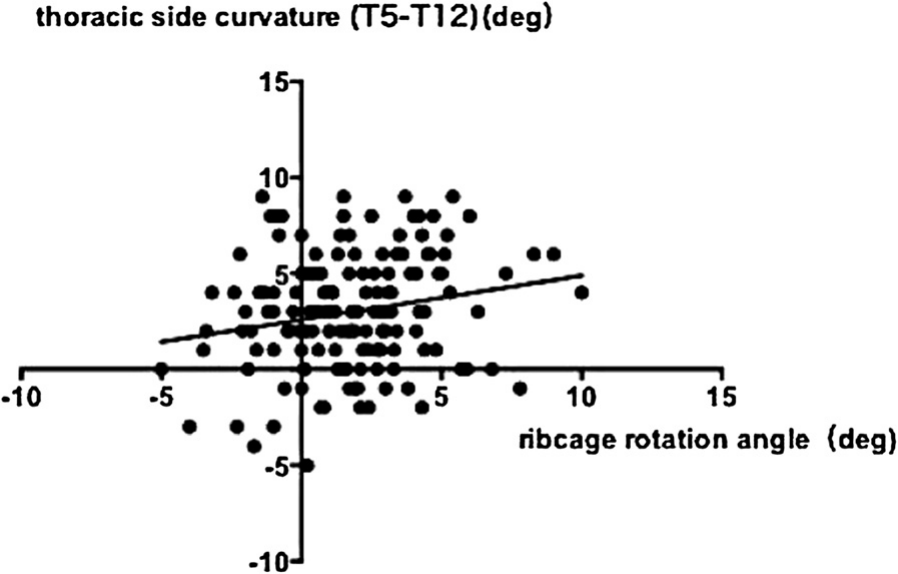
**Correlation between the ribcage rotation angle and the thoracic side curvature.** There was a statistically significant correlation between the ribcage rotation angle and thoracic side curvature (ρ = 0.175, p = 0.033, Spearman’s rank correlation coefficient).

The thoracic side curvature (T5 to T12) was 3.0 ± 3.0 degrees (mean ± SD) (Figure 2). The anteroposterior chest dimension was 83.8 ± 10.8 mm (mean ± SD), the aortic position was 15.1 ± 6.6 mm (mean ± SD) and the rib cage rotation was 1.8 ± 2.6 degrees (mean ± SD) in the normal spine.The correlations between the anteroposterior chest dimension, aortic left shift, rib cage rotation and thoracic side curvature were analyzed. Among these variables, there were significant correlations between the anteroposterior chest dimension and aortic left shift (ρ = 0.53, p < 0.0001) (Figure 3), aortic left shift and rib cage rotation (ρ = -0.284, p = 0.0005) (Figure 4) and rib cage rotation and thoracic side curvature (ρ = 0.175, p = 0.033) (Figure 5). The correlation between the anteroposterior chest dimension and thoracic side curvature (ρ = -0.1427, p = 0.0836) and the correlation between the aortic position and thoracic side curvature were not significant (ρ = 0.1491, p = 0.0706).

## Discussion

Right thoracic curvature, rib cage rotation and thoracic vertebral right rotation have been reported to become dominant after adolescence in the normal spine [9,12]. The prominence of the right scapula in the normal spine is reported to correspond to right thoracic rotation. In the present study, the significant correlation between a shallow chest and the aortic position were confirmed. These deformities are similar to those observed in patients with AIS [7].

The presence of a relationship between organ anatomy and thoracic deformities, such as vertebral rotation and thoracic side curvature, is suggested by observations in humans with situs inversus totalis [10,14]. In patients with situs inversus, thoracic vertebral rotation and thoracic curvature occur in reverse directions. There is a hypothesis that the position of the heart and aorta may be the cause of scoliosis [15-17]. The present findings of a relationship between the aortic position in a shallow chest and right thoracic curvature in the normal spine support the interpretation that a shallow chest and aortic left shift are associated with the initiation process of small thoracic curvatures and possibly also of right thoracic AIS.

Cole AA et al. reported that a decreased AP chest diameter may be a risk factor for the development of AIS by altering the spinal mechanics during movement [5,6]. We recently reported that disturbance of ribcage development results in progressive thoracic scoliosis in mice [8]. The mouse model also demonstrated that the position of the heart is a significant factor that influences the direction of thoracic curvature, thus indicating that the imbalanced load to the vertebral body is derived from an indirect mechanical effect travelling through the ribs on both sides owing to the asymmetry of the position of the heart in the limited thoracic cavity [8].

We examined a series of CT scans of patients who visited our institution for investigating other diseases (Table 1). To eliminate the effects of each disease, it is ideal to study healthy volunteers; however, this is difficult due to the risk of irradiation. Furthermore, one of the limitations of this study may be associated with the fact that these findings represent a snapshot in time in mature subjects with minimal curves. In order to evaluate the potential development of AIS, it is ideal to obtain cross-sectional data using thoracic CT scans in healthy juvenile volunteers and follow the time course of the deformities. Such studies are also difficult to performed due to ethical issues.

Kouwenhoven JW et al. previously reported that there are 2.365 degrees of T8 vertebral right rotation in the normal spine [11]. We described the aorta as having a left shift in this study. There may be an argument that the aortic left shift is an only the comparative position because of the right shift of the vertebra. Our measurements using the rib head as a baseline measured the left-posterior direction, and the aortic position ranged from -3.0 mm to 31.8 mm relative to the rib head, and those values are considered to be larger than the right shift of the vertebrae in the normal spine. Furthermore, in a previous study regarding preoperative non-congenital spines [7], shallow chest significantly correlated with an aortic leftward shift (reference 7, Figure 5). We therefore believe that the influence of the right vertebral rotation was relatively minor, if any at all, and the main cause of the aortic position is thus believed to be the aortic left shift.

## Conclusions

There was a significant correlation between a shallow chest and the aortic position, between the aortic position and the rib cage rotation and between the rib cage rotation and the thoracic side curvature in the normal spine. The findings support the interpretation that a shallow chest and aortic left shift are associated with the initiation process of small thoracic curvatures and possibly also of right thoracic AIS. These factors may not be causal for right thoracic AIS.

## Competing interests

The authors declare that they have no competing interest.

## Authors’ contributions

TD has contributed to conception and design of the study, acquisition of data, analysis and interpretation of data, and drafting the manuscript. YM, OT and KT performed part of acquisition of data. SO, KK, KH and MH performed part of literature review. YI participated in design and coordination and helped to draft the manuscript. All authors read and approved the final manuscript.
